# Probing the Local Environment in Potassium Salts and Potassium-Promoted Catalysts by Potassium Valence-to-Core X-ray Emission Spectroscopy

**DOI:** 10.1021/acs.inorgchem.4c02069

**Published:** 2024-08-20

**Authors:** Atanu Rana, Sergey Peredkov, Malte Behrens, Serena DeBeer

**Affiliations:** † Max Planck Institute for Chemical Energy Conversion, Stiftstraβe 34-36, D-45470 Mülheim an der Ruhr, Germany; ‡ Institute of Inorganic Chemistry, Kiel University, Max-Eyth-Str. 2, 24118 Kiel, Germany

## Abstract

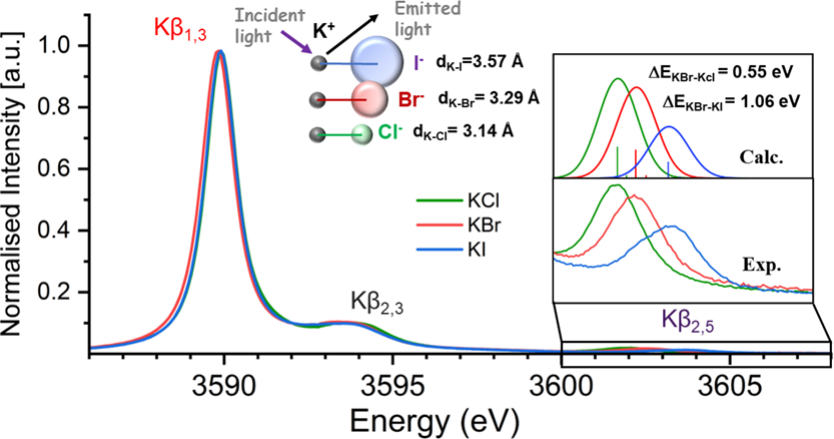

Potassium plays an important role in biology as well as a promoter in heterogeneous catalysis. There are, however, limited characterization techniques for potassium available in the literature. This study elucidates the potential of element-selective X-ray emission spectroscopy (XES) for characterizing the coordination environment and the electronic properties of potassium. A series of XES measurements were conducted, primarily focusing on the VtC transition (Kβ_2,5_) of potassium halides (KCl, KBr, and KI) and oxide-bound potassium salts, including potassium nitrate (KNO_3_) and potassium carbonate (K_2_CO_3_). Across the series of potassium halides, the VtC transition energy is observed to increase, as accurately reproduced by TDDFT calculations. Molecular orbital analysis suggests that the Kβ_2,5_ transition is primarily derived from halide *n*p contributions, with the primary factor influencing the energy shift being the metal–ligand distances. For oxide ligands, an additional Kβ″ transition appears alongside the Kβ_2,5_, which is attributed to a low-energy ligand *n*s, as elucidated by theoretical calculations. Finally, the XES spectra of two potassium-promoted catalysts for ammonia decomposition/synthesis were measured. These spectra show that potassium within the catalyst is distinct from other K salts in the VtC region, which could be promising for understanding the role of potassium as an electronic promoter.

## Abstract 2

Herein, through a combination of experiment and theory, we have established potassium (K) valence-to-core (VtC) X-ray emission spectroscopy (XES) as a tool to probe the coordination environment of potassium. A comprehensive calibration study on various potassium salts has been performed to determine the informational content of K XES. Finally, K XES is applied to a potassium-promoted iron ammonia synthesis and decomposition catalyst to evaluate the form of potassium within the catalyst.

## Introduction

Potassium plays an essential role in countless biological and chemical processes.^1,2^ Notable examples include the role of K^+^ in regulating ion channels,^3−5^ which control a diverse array of cellular processes,^6,7^ including the function of neuronal, muscular, and cardiac tissue.^8^ In chemistry, potassium serves as an electronic promoter in heterogeneous catalysts, where it is essential for obtaining optimal activity in Fischer–Tropsch, Haber-Bosch catalysts, and reverse water gas shift (RWGS) catalysts.^9−15^ Despite the wide range of important processes in which potassium is involved, experimental probes for K ions are relatively limited. K ions are not visible from optical absorption, and the [Ar] electron configuration of the cation also renders it EPR silent. In biology, most of our present understanding of potassium coordination environments has come from protein crystallography.^7,16,17^ More recently, developments using magic angle spinning ^39^K NMR have shown promise as an alternative method for the characterization of the local K environment.^18,19^ Further, we note that element-selective probes, such as X-ray absorption (XAS) and XES, should, in principle, provide another means to selectively characterize the changes that occur in the potassium coordination environment. While several reports have been made using potassium extended X-ray absorption fine structure (EXAFS),^21,22^ to our knowledge there are only a few previous reports of K XES spectroscopy, and to date, reports have focused only on potassium halides (KCl, KBr, and KI).^20−22^ The relative sparsity of both XES (and XAS/EXAFS) studies on K is, in our view, due in large part to the limited number of synchrotron beamlines where such measurements can be carried out.^23−27^ Recently, we have designed and commissioned the PINK beamline at BESSY, which is an ideal setup for XES in the tender X-ray regime,^28^ thus enabling measurements on potassium and opening up the possibility for K measurements on catalytic systems. Of particular interest in this context are Kβ and valence-to-core (VtC) XES spectra, which have in recent years seen widespread use for a range of first row transition metals due to the now well-established sensitivity to ligand identity and protonation state.^29−38^ In contrast, the applications of XES to alkali and alkali earth metals have been very limited,^39,40^ and to our knowledge, no applications of K XES to catalytic materials have been previously reported.

Herein, we perform a systematic calibration study on a series of potassium salts in order to establish the information content of Kβ and VtC XES. The sensitivity of the K VtC XES to ligand identity and potassium-ligand distance is established. Our experimental results are correlated to density functional theory (DFT) calculations in order to more quantitatively assess the spectral changes. The potential of K VtC XES for future studies in chemical catalysis is highlighted through application of this method to a potassium-promoted iron ammonia synthesis catalyst.^41,42^

## Materials and Methods

### Sample Preparation

Potassium chloride (KCl), potassium bromide (KBr), potassium iodide (KI), potassium nitrate (KNO_3_), and potassium carbonate (K_2_CO_3_) were purchased from Sigma-Aldrich and used without further purification.

Fe/MgO catalysts were synthesized from MgFe_2_O_4_ precatalysts, which were obtained from Fe/Mg layered double hydroxide (LDH) precursor samples based on a previously reported procedure,^41,42^ and promoted by impregnation with KNO_3_. The following commercially available chemicals were used without further purification: iron(II) sulfate heptahydrate (≥99.5% p.a., ACS, Carl Roth GmbH & Co. KG), iron(III) nitrate nonahydrate (≥98% p.a., ACS, Alfa Aesar GmbH), and magnesium nitrate hexahydrate (≥98%, ACS, Alfa Aesar GmbH). The precipitation agent was a mixture of sodium carbonate (p.a., AppliChem GmbH) and sodium hydroxide (≥99%, VWR International BVBA). For wet-impregnation, potassium bicarbonate (puriss., Riedel-de Haën) was used without further purification. The synthesis of the LDH phase was conducted through pH-controlled coprecipitation at pH = 10.5 and 50 °C using an automated OptiMax synthesis workstation provided by Mettler Toledo. A solution with a 1:1:1 ratio of Mg^2+^, Fe^2+^, and Fe^3+^ and a total metal cation concentration of 0.8 mol/L was codosed with the precipitating agent (0.60 mol/L Na_2_CO_3_ and 0.09 mol/L NaOH) to keep the pH constant. The precipitate was aged for 1 h in the mother liquid, washed, recovered by centrifugation, and dried at 80 °C to yield the LDH precursor.

The Catalyst_K_1_ and Catalyst_K_2_ were prepared using two distinct methods, which varied depending on the addition steps of the promoter. In both samples, the mass of KNO_3_ was adjusted to yield a K loading of 2 wt %. In the preparation of Catalyst_K_1_, KNO_3_ was impregnated onto the LDH precursor prior to the calcination step, whereas for Catalyst_K_2_, the LDH precursor was first calcined, followed by impregnation of KNO_3_ and subsequent recalcination. The calcination process was carried out in a muffle furnace at 600 °C (with a heating rate of 2 K/min) for 2 h. Calcination led to the decomposition of the LDH structure and the formation of a K-promoted MgFe_2_O_4_ spinel. In the unpromoted reference sample^39^ as well as in Catalyst_K_2_, an α-Fe_2_O_3_ (hematite) byphase was detected. Subsequent to catalyst preparation, the catalytic activity was tested in both instances.

All samples were finely ground and pressed into pellets. Potassium salts were sealed using 30 μm Kapton tape windows. Samples containing iodide and bromide were additionally shielded with 800 nm Al foil to prevent detector interference from visible light fluorescence. For ammonia synthesis catalysts (Catalyst_K_1_ and Catalyst_K_2_), 8 μm Kapton tape served as a protective cover. The catalysts were studied in their calcined state. Subsequently, the samples were transferred into the spectrometer’s vacuum system and measured at room temperature.

### XES Measurements

All potassium Kβ X-ray emission spectroscopy (XES) data were collected at the PINK tender X-ray beamline at BESSY II. The beam size was 30 × 500 μm fwhm (*V* × *H*). The excitation energy was 4000 eV, and the incoming photon flux was ∼10^13^ ph/s. The fwhm at this excitation energy is 80 eV. The spectra were recorded by the use of a dispersive in vacuum von Hamos spectrometer equipped with a Si(110) cylindrically bent crystal (bending radius *R* = 250 mm) and a Great Eyes CCD (26 μm × 26 μm pixel size, 256 × 1024 pixels) detector. The Si(220) reflection was utilized at Bragg angles from 62 to 65°. Under these conditions, the spectrometer resolution was about 0.8–1 eV.^28^

Calibration of the energy scale was done by measuring the emission lines of the Sb foil. The energy points used for the energy calibration were Sb Lα1:3604.72 eV and Lα2:3595.32 eV (X-ray data booklet).^43^ To define the peak positions, these lines were fitted with split Voigt functions^44^ (an asymmetric shape of La1,2 lines is a sum of natural line and instrumental function asymmetries), in which the width of the distribution is different between left and right slopes. Energies were translated into Bragg angles, and a fit with a tangential function was applied.

### Computational Details

All calculations were performed using the ORCA program package version 5.0.1.^45^ DFT calculations were performed using the BP86^46^ functional in the def2-SVP basis set.^47,48^ All calculations were carried out using the crystallographically reported coordinates without additional optimization.^49−55^ For infinite lattice compounds, calculations were performed by either (1) using a minimum quantum cluster (small QC) comprised of the potassium and its nearest neighbors, a total of 7 atoms, or (2) embedding the larger quantum cluster (large QC), a total of 80 atoms. For larger quantum cluster formation, the “Crystal Prep” technique^56^ has been used, which is available in the present version of ORCA. It was determined that the minimum quantum cluster approach more closely agreed with experimental trends as compared to the embedding quantum cluster method. Therefore, calculations utilizing solely the minimum quantum region are the primary focus. Calculated VtC XES spectra are based on the combined electric and magnetic dipole and electric quadrupole contributions.^29,57,58^ However, only electric dipole transitions were found to have non-negligible intensities. The calculated energy axis is not an absolute energy axis. To make our comparison more reasonable, the calculated energy axis is shifted by a constant value of 104.4 eV to align with the experimental energy axis. Reported spectra are Gaussian broadened by 2.0 eV. This broadening was chosen in order to obtain the best visual agreement between the calculated and experimental spectra. The broadening accounts for the spectrometer resolution and the K 1s core hole lifetime as well as additional factors such as site heterogeneity and possible charge transfer and/or multielectron excitations, which also contribute to the experimental broadening.

## Results and Analysis

### Potassium Halide Salts

**Figure 1:**
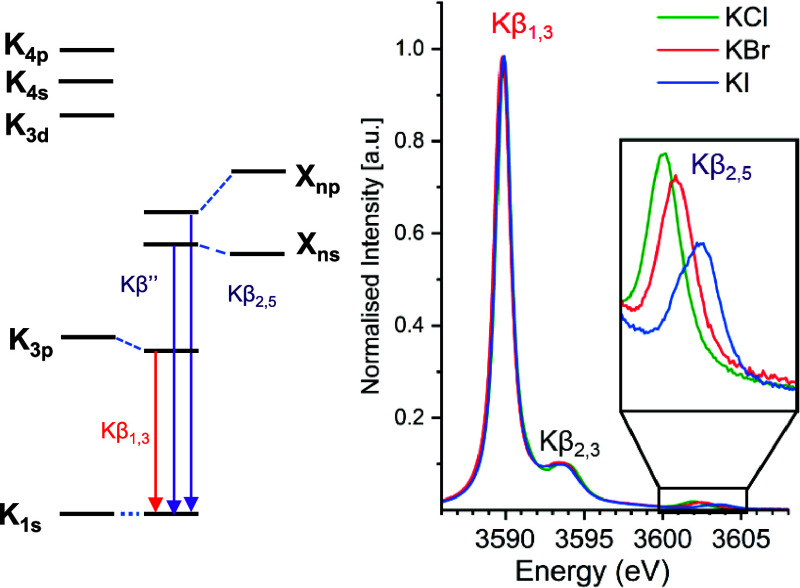
Simplified MO diagram schematically depicting the origin of the transitions in the K Kβ XES spectrum (left) and the experimental XES spectra of KCl, KBr, and KI, with the VtC region enlarged approximately 50-fold in the inset (right). The Kβ XES spectral intensities were normalized to 1.0 at the maximum of the Kβ_1,3_ mainline.

Figure 1 (right panel) compares the Kβ mainline for KCl, KBr, and KI. All spectra show a maximum at ∼3589.7 eV, which corresponds to a 3p–1s transition (Figure S1). The similar energy for all potassium salts is consistent with the expected similar K description in all compounds. Only the Kβ main line of KBr is ∼0.1 eV lower than others, which might arise due to some ligand-derived contribution to this transition. In addition, all of the potassium salts show a shoulder at ∼3593.8 eV. The 4 eV splitting is clearly too large to be attributed to K 3p spin-orbit splitting and may potentially result from a two-electron process. We note that this feature was also observed in the previously reported Kβ spectrum of potassium halide salts, where it was assigned as a satellite feature resulting from a double ionization event.^25,26^ We note that for all five potassium samples measured in this study, the Kβ main line does not vary by more than 0.1 eV (Figure S1). Hence, for the remaining compounds, we focus on the high-energy VtC features, which show clear variations across the series.

**Figure 2:**
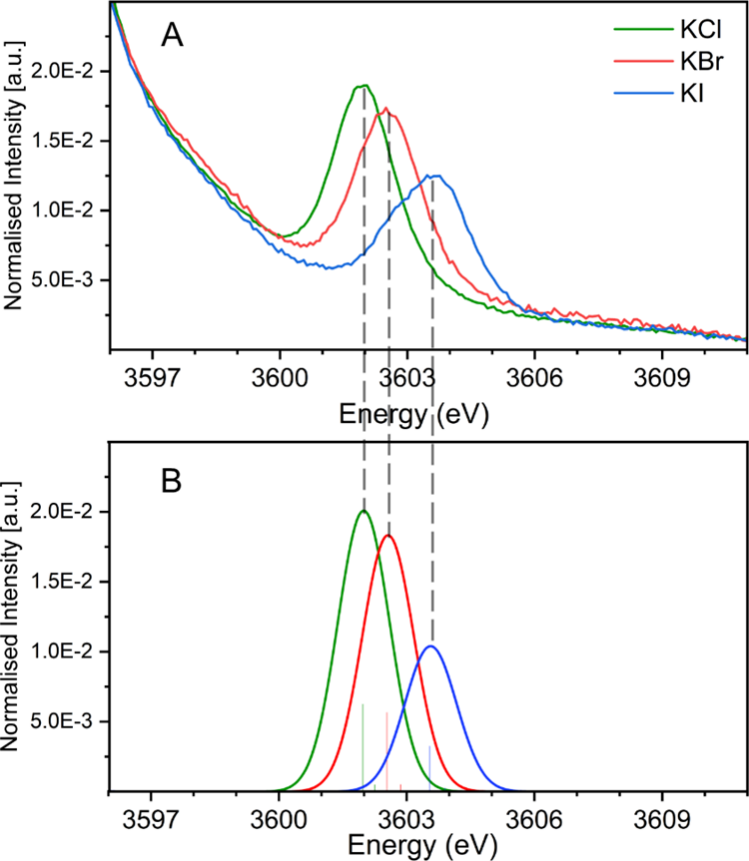
(A) Experimental and (B) calculated spectral comparison of the VtC XES region of KCl, KBr, and KI. The DFT calculated energy axis is shifted by 104.4 eV.

Figure 2A depicts the VtC XES images for the potassium halides. Based on analogy to transition-metal VtC XES, the observed features may be attributed to transitions from the filled halide *n*p valence levels to the K 1s core hole (or so-called Kβ_2,5_ features). Ongoing from KCl to KBr to KI, these features shift up in energy by ∼1.6 eV (Table 1). This is consistent with a decrease in the ligand valence ionization energy. We note, however, that the valence ionization energies change by only 0.55 eV on going from Cl^–^ to I^–^ and hence the difference in ionization energy is not the only source of the observed shift.^59^

**Table 1:** Experimental and Calculated Energy of Kβ_1,3_ and Kβ_2,5_ Transitions^a^

electronic transition		KCl	KBr	KI
Kβ_1,3_(eV)	experimental	3589.9	3589.81	3589.91
	calculated	3592.05	3592.05	3592.13
Kβ_2,5_(eV)	experimental	3601.9	3602.5	3603.62
	calculated	3602.00	3602.56	3603.58

a The calculated energy axis is shifted 104.4 eV.

In order to more quantitatively understand the origin of the modulations in energy and intensity of the Kβ_2,5_ features across the halide series, DFT calculations were performed. The calculations were carried out in two different ways: (i) calculating the larger quantum cluster (large QC includes 80 atoms per calculation) and (ii) considering a minimum quantum cluster composed of a [KX_6_]^5–^ unit (Figure S2). As shown in Figure 2B, the calculated XES spectra with minimal QC cluster successfully reproduce the relative energy and intensity trends for the Kβ_2,5_ features of all of the halides. We note, however, that in the experimental VtC spectrum for KI and KBr, there is an asymmetry in the Kβ_2,5_ features that is not captured by our calculations. These may arise from the energy differences between the halides (Br^–^ and I^–^) in their *P*_1/2_ and P_3/2_ states, as established by Gomes and co-worker using relativistic embedded equation of motion coupled cluster theory.^60^ However, the level of theory employed in this study is not sufficient to capture such effects. The embedded QC calculations somewhat underestimate the shifts among different complexes. This may suggest that there is too much charge compensation from the lattice (Figure S3C). Given the already good agreement with a minimal quantum cluster, all further analyses have thus been performed considering only the small QC calculations in our study.

The calculated spectra were then analyzed in terms of the transitions shown in Figure 1. The transitions are Kβ_1,3_ (3p to 1s), Kβ″ (ligand *n*s to K 1s), and Kβ_2,5_ (ligand *n*p to 1s) accordingly (Figures S4–S6). We note in the present case, both experimentally and computationally, there is significant overlap between the Kβ_1,3_ and Kβ″ features. Hence the orbital population analysis is given in terms of K 3p, L *n*s, and L *n*p contributions. In the Kβ_1,3_ region, the K 3p character dominates in all cases (>92% for KCl and KI, ∼73% for KBr), while in the Kβ_2,5_ region, ligand p contributions dominate (>92%, Table S2). However, the low-intensity Kβ″ features are not well resolved for the halides, as these appear to largely overlap with the Kβ_1,3_. The orbital analysis shows that the relative energy of the ligand *n*s orbital decreases from I^–^ to Br^–^ to Cl^–^. The Br^–^*n*s orbital has an energy level closest to K 3p, resulting in a higher degree of orbital overlap. Consequently, the Br *n*s character (25%) significantly increases in the Kβ_1,3_ region relative to the other two halides.

The experimental and calculated VtC XES spectra are compared in Figure 2B and show that the trends in both energies and intensities are well reproduced at this level of theory. As ionization energy differences can account for only a portion of the observed shift, additional calculations were performed in order to understand the impact of potassium-halide distance on the VtC energies as well as their intensities. As is expected, the K-halide distance increases across the series going from *d*_K–Cl_: 3.14 Å to *d*_K–Br_: 3.29 Å to *d*_K–I_: 3.53 Å, due to the difference in the ionic radius of the corresponding halides. Therefore, in addition to the intrinsic ionization energy contributions, the K-halide distance may also play a role in the observed Kβ_2,5_ energies and intensities. To systematically test this, calculations were performed by changing the average potassium-halide distances from 3.0 to 3.6 Å with a periodic increment of 0.2 Å.

**Figure 3:**
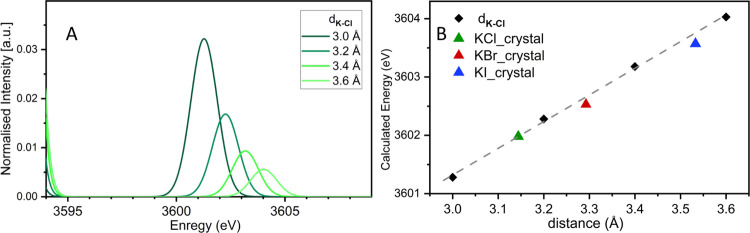
(A) Calculated K VtC XES region for KCl for varying K–Cl distances from 3.0 to 3.6 Å. (B) Correlation of K-halide distances with the Kβ_2,5_ transition energies. The colored triangles correspond to the experimental distances, while the black diamonds correspond to the hypothetical distances from panel A. The DFT calculated energy axis is shifted by 104.4 eV.

As shown in Figure 3A,B, the Kβ_2,5_ transition energy increases linearly with increasing K–Cl bond length, while the peak intensity correspondingly decreases. These trends are consistent with the bonding molecular orbital being less stabilized (hence, the higher energy) and having less potassium character (hence, the decreased intensity). A similar trend is also observed for KBr and KI (Figure S7) with a similar distance-dependent energy shift and decrease in intensity upon elongation of the K–X bond. Figure 3B shows an overlay of the calculated KCl, KBr, and KI Kβ_2,5_ transition energies at the experimental K-halide distances (green, red, and blue triangles for KCl, KBr, and KI, respectively) as compared to the calculated KCl Kβ_2,5_ energies for K–Cl distances from 3.0 to 3.6 Å (black diamonds). The black line is a fit through the points generated from the hypothetical K–Cl series. The comparison plot indicates that the modulations in the energy of the Kβ_2,5_ features across the halide series mainly originate from the inherent metal-ligand bond distances of the corresponding potassium halide. The slight deviations of the KBr and KI from the K–Cl distance-dependent trend line are likely a reflection of the differences in ionization energy of Br and I relative to Cl. These shifts of 0.13 and 0.20 eV for KBr and KI relative to the trend line are in good agreement with the relative changes in the Br and I ionization energies relative to that of Cl (0.25 and 0.55 eV, respectively). It has been observed that the VtC transition intensity decreases ongoing from KCl to KI both in experimental and theoretical calculated results. A similar trend is observed in calculated results when increasing the hypothetical K–X (X = Cl, Br, I) distance. These observations indicate that the K–X distance also plays a significant role in the transition intensity. To further investigate the effect of ligand identity on this transition intensity, we have compared KCl and KBr Kβ_2,5_ intensities at a K–X distance of 3.2 Å, and a significant difference in the intensity ratio was observed (Intensity ratio (*I*_KBr/KCl_) = 1.39). A noticeable difference for all the halides is the different atomic radii (1.81, 1.96, and 2.2 Å for Cl^–^, Br^–^ and I^–^, respectively), an indication of different polarizabilities (3.69, 4.81, and 7.16 Å^3^ for Cl^–^, Br^–^ and I^–^, respectively).^61^ The polarizability ratio between Br^–^ and Cl^–^ is 1.31, similar to the difference in Kβ_2,5_ intensity ratios, as noted above. These results indicate that the VtC Kβ_2,5_ intensity depends on both the metal-ligand distance and the ligand polarizability.

#### KNO_3_ vs K_2_CO_3_

**Figure 4:**
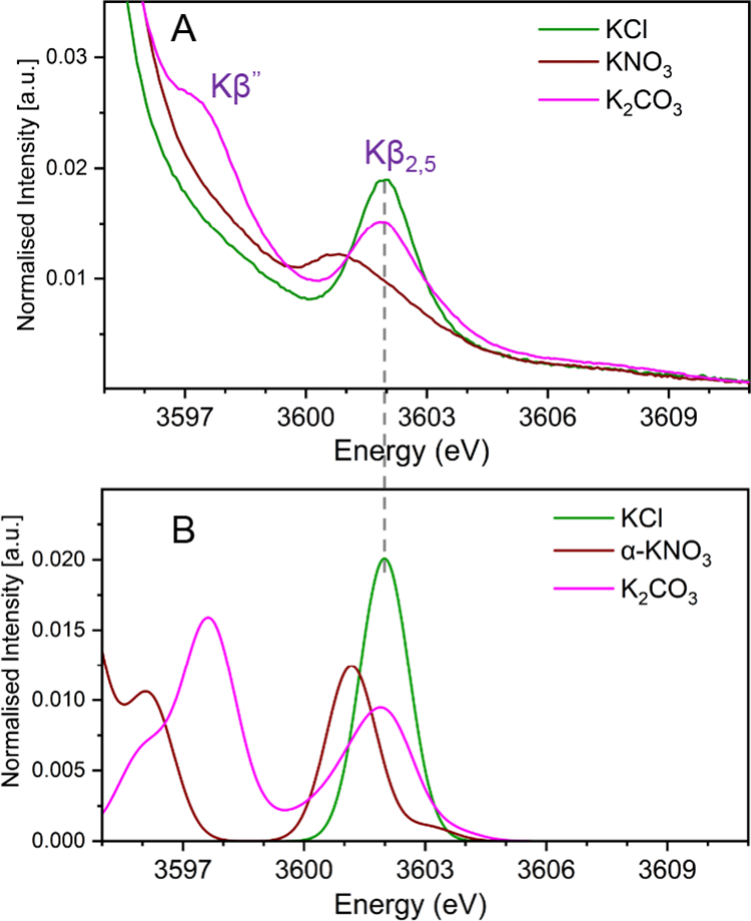
(A) Experimental and (B) calculated spectral comparison of the VtC XES spectra of KCl, KNO_3_, and K_2_CO_3_. The DFT calculated energy axis is shifted by 104.4 eV.

Having established trends in the VtC XES energies and intensities for potassium halides, we then extended this approach to oxygen-bound potassium complexes, including K_2_CO_3_ and KNO_3_. The results showed that the Kβ_1,3_ transitions of K_2_CO_3_ and KNO_3_ occur at energies similar to those of the potassium halides, around 3589.7 eV (Figure S1). Figure 4A depicts an overlay plot of the experimental VtC XES region of K_2_CO_3_ and KNO_3_ with KCl, included as a reference halide for comparison. The highest energy VtC feature for K_2_CO_3_ is at a similar energy to that of KCl (3601.9 eV for K_2_CO_3_ vs 3601.97 eV for KCl), albeit lower in intensity. Further K_2_CO_3_ exhibits an additional VtC transition at 3597.43 eV in the region typically referred to as the Kβ″ region. In contrast for KNO_3_, only a single Kβ_2,5_ transition appears at 3600.79 eV, which is ∼1.1 eV lower than that of KCl and K_2_CO_3_.

Theoretical calculations were performed to understand these transitions. The experimental XES spectrum was measured using a powder form of potassium salts. However, the crystal structures of potassium salts in theoretical calculations can be a useful starting point for a logical initial guess. For our calculation, the structural coordinates have been taken from the reported crystal structures.^52,54,55^

The K_2_CO_3_ has two inequivalent potassium coordination sites in its crystal structure. One site is six-coordinated with six monodentate carbonate ligands, while the other is nine-coordinated with five carbonate ligands (one monodentate and four bidentate, Figure S8C). The calculated Kβ X-ray emission spectrum of K_2_CO_3_ contains both high-energy Kβ_2,5_ and low-energy Kβ″ features. This is in good agreement with the experimental observations (Figure 4). The orbital population analysis of the calculated spectrum indicates that the Kβ″ transition arises from the hybridization of K 3p orbitals with O 2s valence orbitals. The lower the energy of the Kβ″ transition, the greater the contribution from O 2s orbitals. To deconvolute the individual contributions of the two potassium coordination sites to the VtC spectrum, two separate calculations have been performed (Figure S9B). The results show that both sites contribute to the Kβ_2,5_ region, but the nine-coordinated site [K(CO_3_)_5_] dominates the spectral intensity in this energy region. In contrast, the six-coordinated site [K(CO_3_)_6_] is the dominant contributor in the Kβ″ region (Figure S9). Notably, in the K_2_CO_3_ crystal structure, the average K–O bond distance of the six-coordinate site is 2.67 Å, and for the nine-coordinate site, it is 2.96 Å. Comparing the coordination-site-specific relative intensities and the average metal-ligand distances, it is clear that shorter K–O distances lead to higher transition intensities.

KNO_3_ is known to exist in four crystal phases: α, β, γ, and delta (δ). The α and δ phases are stable at room temperature, while the β and γ phases are metastable.^53^ The β and γ phases can be induced by heating or cooling the α phase in a certain temperature range. As the current study was carried out at room temperature, the α and δ phases (Figure S9) were explored for the DFT calculations. The results of these calculations are shown in Figure S10. While both phases show a predicted Kβ_2,5_ transition at ∼3601.2 eV, in both cases, the calculated spectrum is ∼0.4 eV higher in energy relative to the experiment. It is possible that the powder form of the KNO_3_ sample introduces some disorder that is not modeled by the DFT calculations of the crystallographic models. We note, however, that the qualitative energy and intensity trends for KNO_3_ relative to KCl and K_2_CO_3_ are reasonably well captured by theory, clearly showing that the Kβ_2,5_ transition of KNO_3_ appears to lower energy with decreased intensity.

#### K-XES Comparison with NH_3_ Synthesis/Decomposition Catalyst

Having assigned the K VtC XES spectra for a series of K salts of known composition, we then extended our K VtC XES to potassium-impregnated MgFe_2_O_4_ catalysts utilized in NH_3_ synthesis and decomposition reactions.^39,62^ In heterogeneous catalysis, small amounts of potassium ions are frequently used as catalytic promoters. In the present case, KNO_3_ was used as a potassium source as described in the materials and method section to result in an approximate potassium loading of 2 wt % (2,05% for Catalyst _K_1_, 1,76% for Catalyst _K_2_ as determined by AAS). Depending upon when the potassium was introduced to the catalysts (before calcination on the LDH precursor or after calcination on the spinel precatalyst), two different types of catalysts have been prepared for our measurements, which are labeled as Catalyst_K_1_ and Catalyst_K_2_. A similar Kβ_1,3_ transition energy (∼3589.7 eV) is observed for both catalysts relative to the potassium salts (Figure S11), again indicating the presence of a similar potassium electronic state.

**Figure 5:**
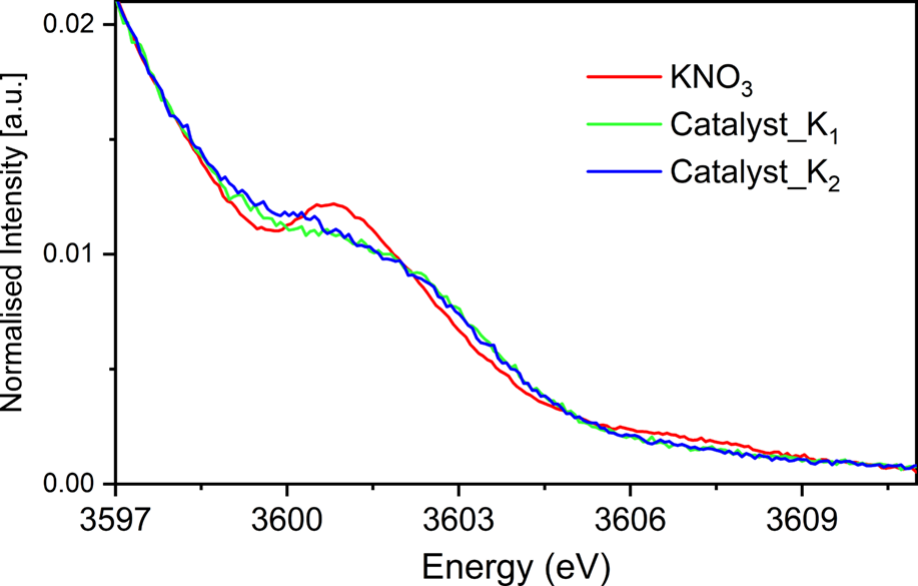
Overlay plot of Kβ_2,5_ transition region of KNO_3_ with the NH_3_ synthesis catalysts Catalyst_K_1_ and Catalyst_K_2_.

Figure 5 depicts an overlay of the VtC XES for the catalysts along with the precursor KNO_3_, which shows weak VtC XES features for both catalysts with a lower intensity and a broader energetic spread relative to the KNO_3_ reference with a slight shift to higher energy. These data suggest that the state of potassium in catalysts after calcination has changed relative to its conformation in the KNO_3_ source material. This can be attributed to the calcination at 600 °C, which triggers the α-to-β phase transition of KNO_3_ at approximately 130 °C, the melting of KNO_3_ at 334 °C, and the beginning of the thermal decomposition into oxygen and potassium nitrite above 550 °C. These temperatures refer to the bulk compound and may differ from those of highly dispersed materials, which can be obtained by the wetting of the catalyst’s pores with a molten salt layer (melt infiltration).^63^

Utilizing the lessons, we obtained from the investigation of the known salts it appears that the potassium is interacting weakly with the surrounding ligands, manifesting in the shift to higher energy and decrease in intensity. In these catalysts, it is anticipated that potassium will predominantly reside on the surface, exhibiting a high dispersion. Consequently, the interaction between potassium and surrounding ligands is expected to be relatively weaker compared to that of bulk species within a crystal lattice. Our findings are consistent with this observation. This modulation in the potassium, however, is the same in Catalyst_K_1_ and Catalyst_K_2_, regardless of the stage of potassium impregnation. While at the present state of understanding, we cannot predict the exact coordination site of potassium in the catalysts, these results suggest that if potassium is directly involved as an electronic promoter during catalysis, this spectral region should provide experimentally observable changes. Hence, the present study provides the groundwork for potential future applications in catalysis.

## Conclusions

This work introduces K XES results for various potassium salts and two potassium-promoted catalysts. A progressive energy shift from KCl to KBr to KI is observed in the Kβ_2,5_ VtC XES transition for potassium halides. Theoretical calculations support the conclusion that potassium-halide distances are the primary factors influencing this energy shift in halide salts. Furthermore, it is confirmed that the Kβ″ transitions for all potassium halides are obscured by the intense Kβ_1,3_ transition. In oxygen-ligated complexes such as K_2_CO_3_, alongside the Kβ_2,5_ transition, an additional Kβ″ transition emerges due to the low-energy contribution from the ligand (O)2s and the short K–O distances, which give rise to increased spectral intensity. The K_2_CO_3_ crystal structure has two inequivalent potassium coordination sites that give rise to distinct features in the VtC XES. The six-coordinated site, with shorter K–O distances, dominates the Kβ″ region, while the nine-coordinated site dominates the Kβ_2,5_ region. This information can be utilized to understand the electronic structure and bonding interactions in K_2_CO_3_. For KNO_3_, while full quantitative agreement is not achieved, the qualitative trends relative to those of other potassium salts are captured. Finally, K XES spectra of an NH_3_ synthesis/decomposition catalyst reveal that the potassium within the catalyst is clearly modulated relative to the KNO_3_ precursor in the VtC region and is also distinct from other K salts. Our understanding at present indicates substantial alterations in the coordination environment of the precursor postcalcination. However, additional research is imperative to precisely forecast the exact potassium configuration within the catalyst. The results presented here form the foundation for future operando studies of catalysts aimed at elucidating the role of potassium as a promoter.

## Data Availability

All relevant data, including the experimental XES data for all potassium salts, NH_3_ decomposition catalysts, and the calculated ORCA files, are available at 10.17617/3.TTWAOX.

## Supporting Information Available

Additional experimental and calculated spectra, as well as transition assignments (PDF)
