# Supplementary material for: Probing the Local Environment in Potassium Salts and Potassium-Promoted Catalysts by Potassium Valence-to-Core X-ray Emission Spectroscopy
Source: Inorg Chem. 2024 Aug 20;63(35):16217–23. doi: 10.1021/acs.inorgchem.4c02069 (PMC11372750; doi:10.1021/acs.inorgchem.4c02069)
Supplement: Supplementary file 1 — ic4c02069_si_001.pdf [file ic4c02069_si_001.pdf]

Supporting Information

Probing the Local Environment in Potassium Salts and Potassium-Promoted Catalysts by Potassium Valence-to-Core X-ray Emission Spectroscopy

AtanuRana,[†] SergeyPeredkov,[†] MalteBehrens,[‡] SerenaDeBeer[*],[†]

[†]Max Planck Institute for Chemical Energy Conversion, Stiftstraβe 34-36, D-45470 Mülheim an der Ruhr, Germany

[‡]Institute of Inorganic Chemistry, Kiel University, Max-Eyth-Str. 2, 24118 Kiel, Germany

[*]Email: serena.debeer@cec.mpg.de.

Atanu Rana - https://orcid.org/0000-0002-2397-5869

Sergey Peredkov - https://orcid.org/0000-0002-5816-9015

Malte Behrens - https://orcid.org/0000-0003-3407-5011

Serena DeBeer - https://orcid.org/0000-0002-5196-3400

Supporting Information:

**Figure S1**	S3
**Figure S2**	S3
**Figure S3**	S4
**Figure S4**	S5
**Figure S5**	S5
**Figure S6**	S6
**Figure S7**	S6
**Figure S8**	S7
**Figure S9**	S7
**Figure S10**	S8
**Figure S11**	S8
**Table S1**	S9
**Table S2**	S9
**Table S3**	S9
**Table S4**	S10
**Reference**	S10

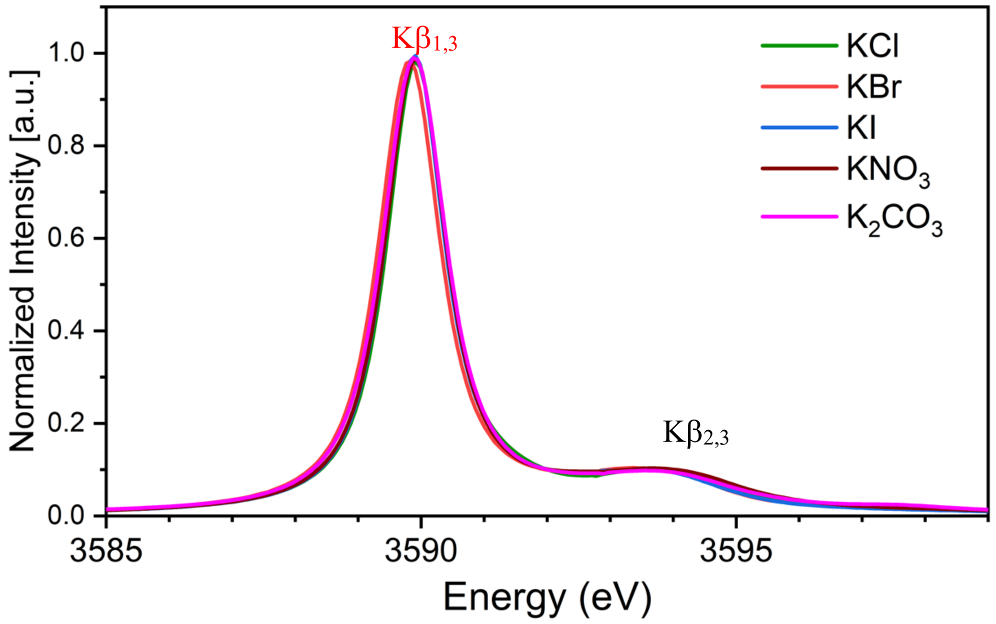

Figure S1. Overlay of experimental K K$\beta_{1,3}$ XES spectra of KCl, KBr, KI, KNO_3, and K_2CO_3.

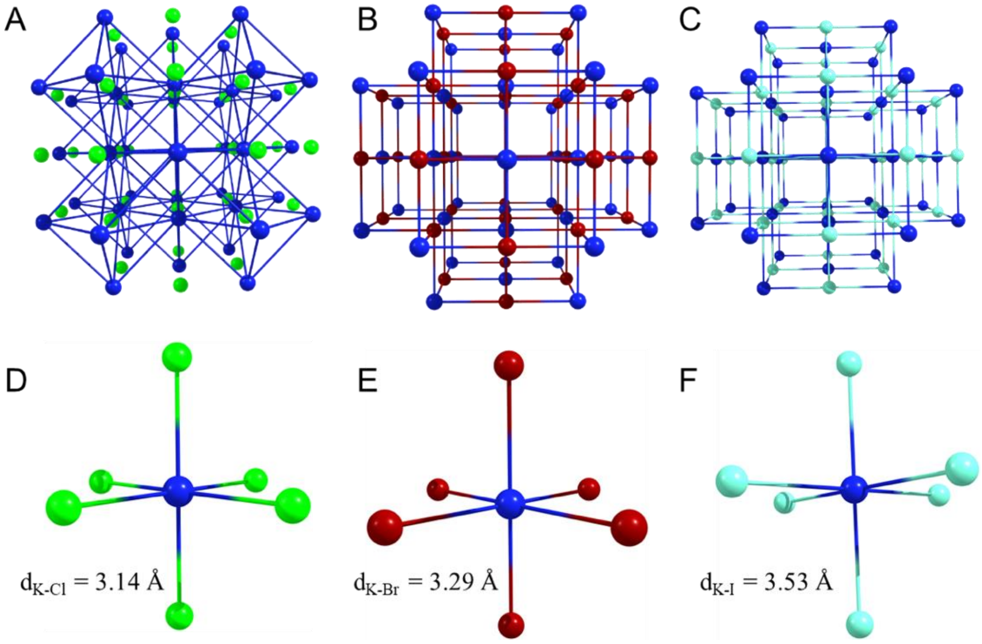

Figure S2. Calculated structural geometries of potassium halides including, KCl, KBr, and KI. A), B), and C) are constructed using crystal prep and D), E), and F) are calculated using a minimum quantum cluster comprised of the potassium and its nearest neighbor.

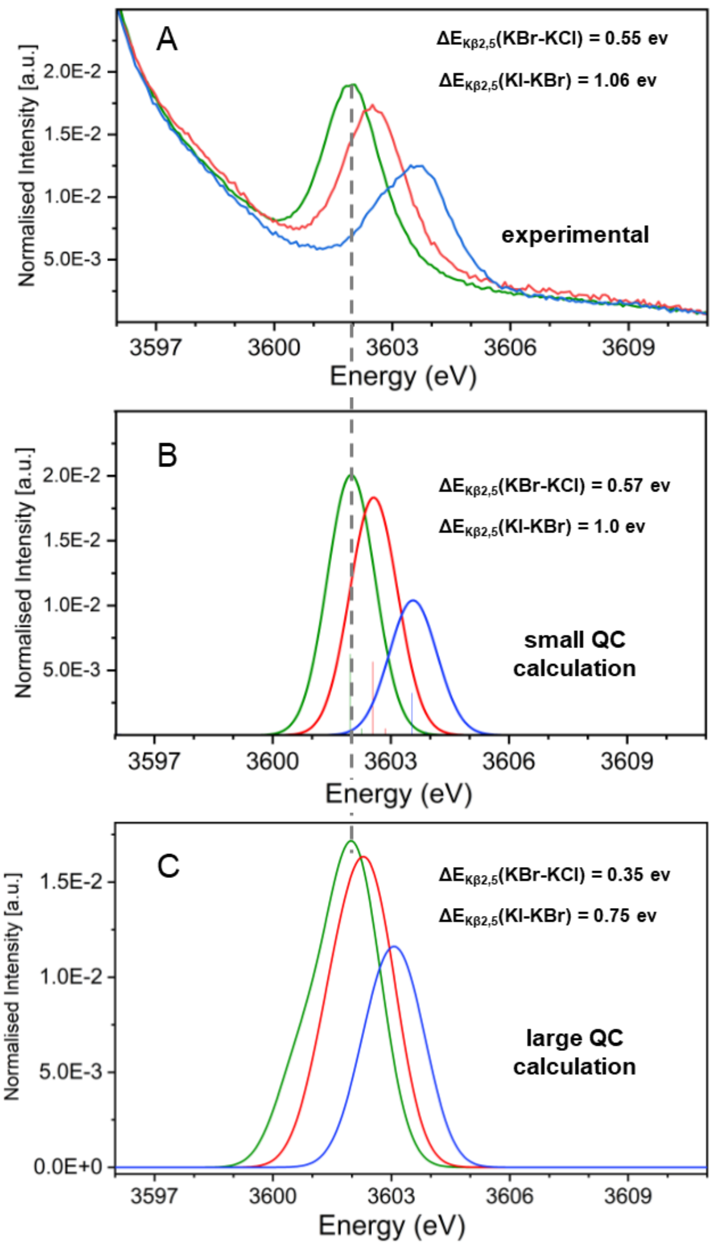

Figure S3. A) Experimental K VtC XES region of KCl, KBr and KI. B) Corresponding calculated spectrum with small QC, and C) calculated spectrum with large QC. The DFT calculated energy axis is shifted by 104.4 eV.

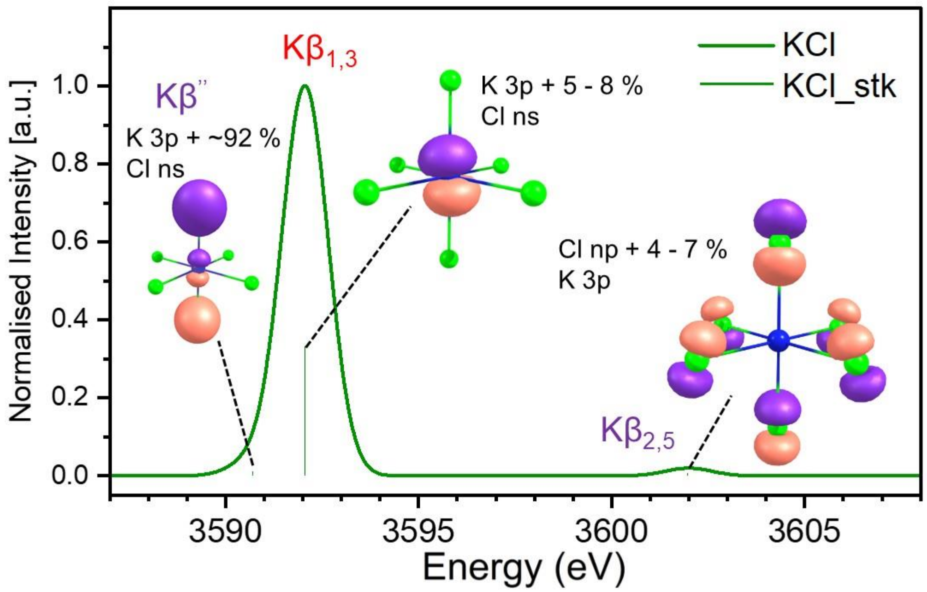

Figure S4. Calculated XES spectrum of KCl with the molecular orbital of K$\beta_{1,3}$, K$\beta_{2,5}$ and Kβ'' transitions. The calculated energy axis is shifted by 104.4 eV.

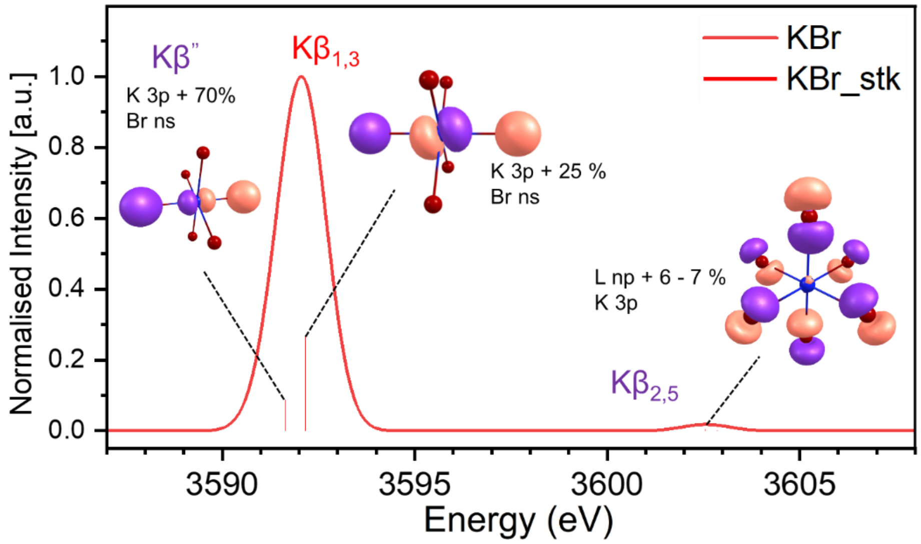

Figure S5. Calculated XES spectrum of KBr with the molecular orbital of K$\beta_{1,3}$, K$\beta_{2,5}$ and Kβ'' transitions. The calculated energy axis is shifted by 104.4 eV.

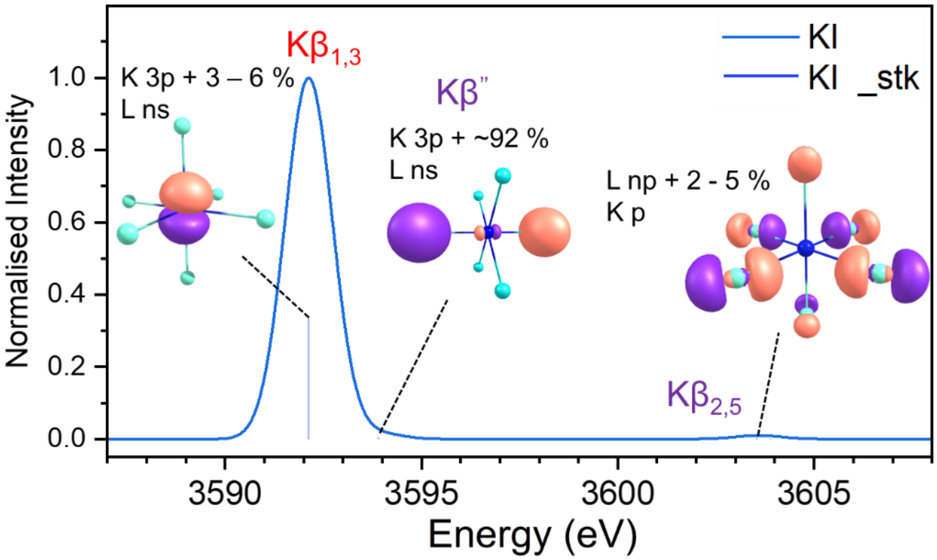

Figure S6. Calculated XES spectrum of KCl with the molecular orbital of K$\beta_{1,3}$, K$\beta_{2,5}$ and Kβ'' transitions. The calculated energy axis is shifted by 104.4 eV.

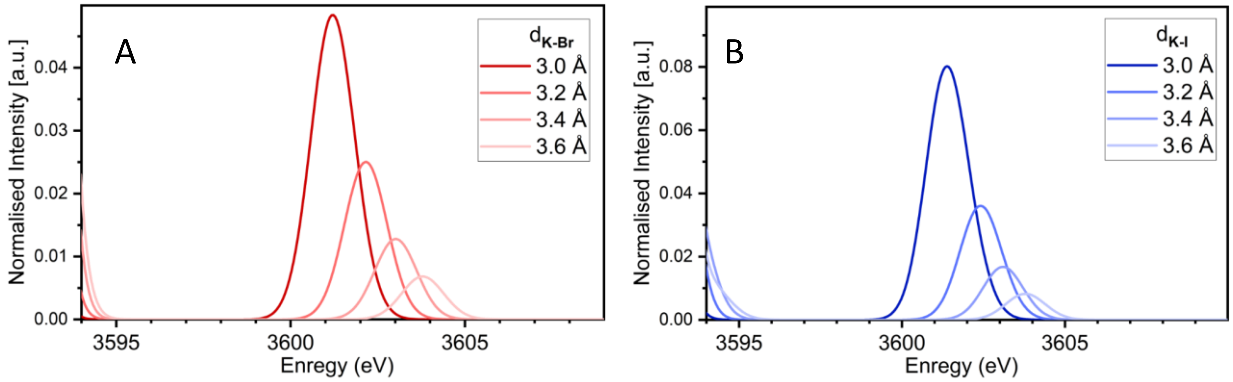

Figure S7. Calculated K VtC XES region of A) KBr and B) KI with potassium-halide distances incremented in 0.2 Å steps from 3.0 to 3.6 Å. The calculated energy axis is shifted by 104.4 eV.

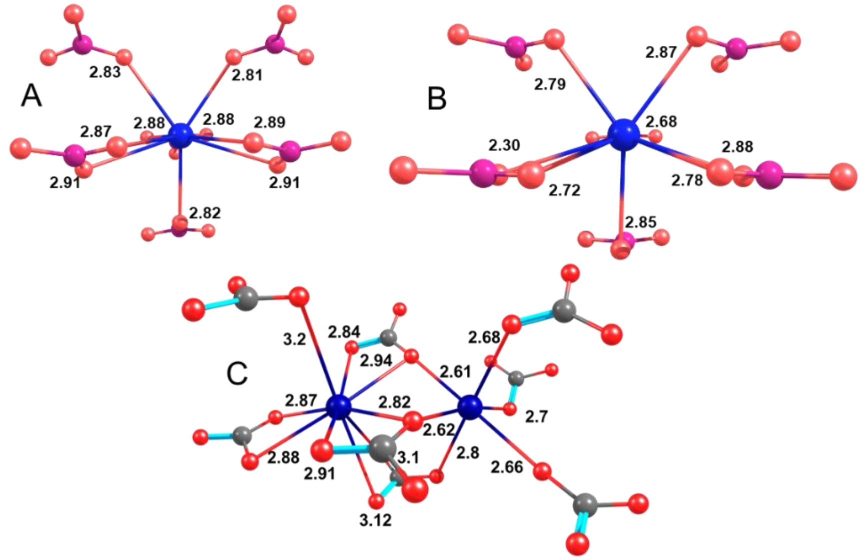

Figure S8. Calculated structures of KNO_3 with two different isomers A) α-phase, B) δ-phase, and C) the structure for K_2CO_3. The coordinates are taken from reported crystal structures.[1-3] All are calculated structures use a minimum quantum cluster comprised of the potassium and its nearest neighbor.

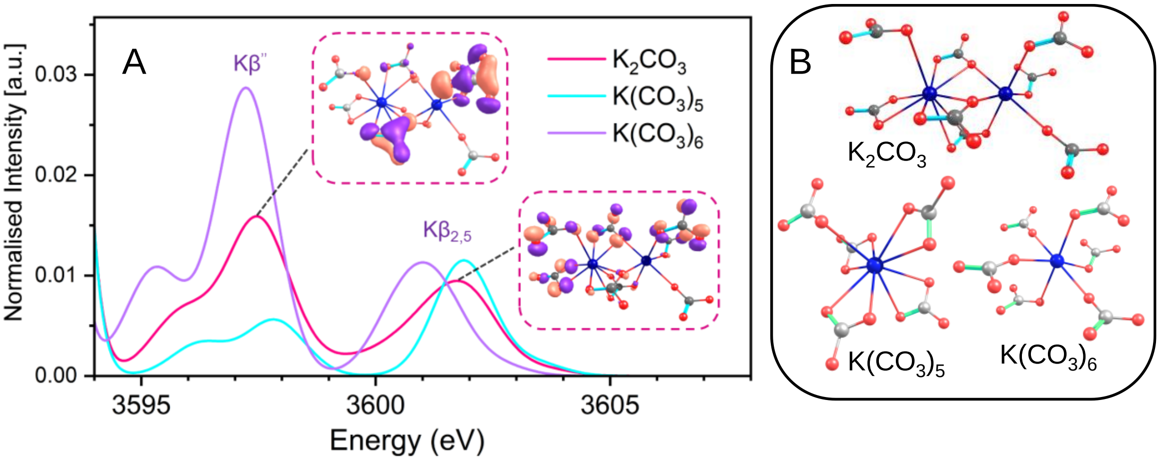

Figure S9. A) Calculated XES spectrum of K_2CO_3 along with the individual K sites: the nine coordinate $K(CO_3)_5$ and the six coordinate $K(CO_3)_6$. B) Calculated structure of K_2CO_3 and deconvoluted nine-coordinate site $K(CO_3)_5$ and six-coordinate site $K(CO_3)_6$. The calculated energy axis is shifted by 104.4 eV.

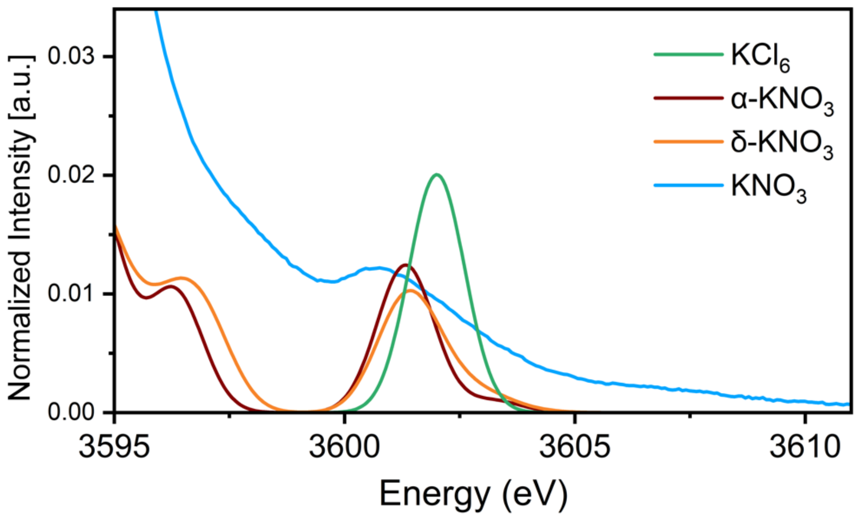

Figure S10. Overlay of calculated K VtC XES region of KCl (green line), α-KNO_3(brown line), and δ-KNO_3(orange line) along with experimental XES spectrum of KNO_3(sky blue line). The calculated energy axis is shifted by 104.4 eV.

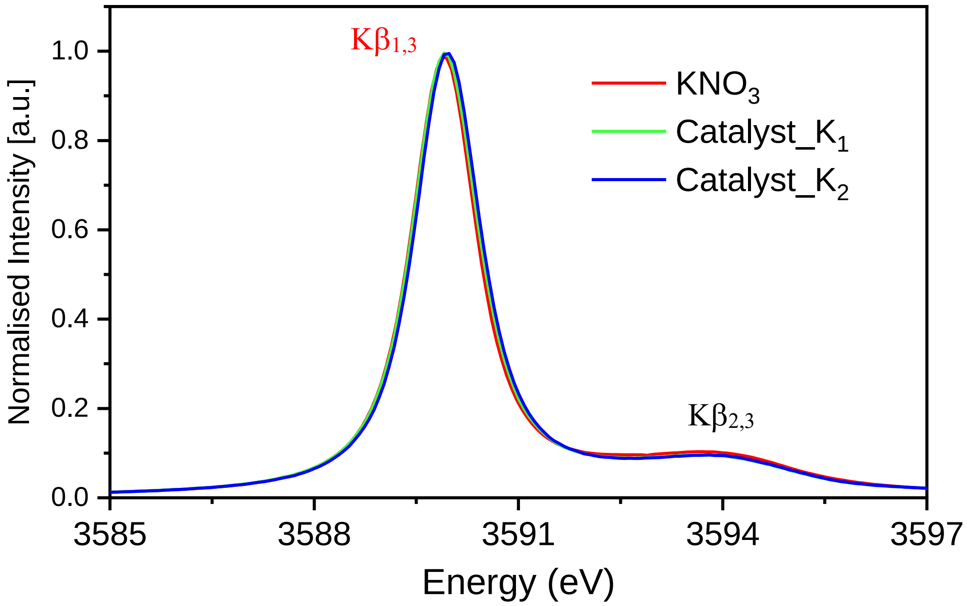

Figure S11. A experimental overlay spectrum of K$\beta_{1,3}$ of KNO_3, Catalyst_K_1, and Catalyst_K_2.

Table S1. Experimental and Calculated Energy of $K\beta_{1,3}$ and $K\beta_{2,5}$ Transitions[a]				
electronic transition		KCl	KBr	KI
$K\beta_{1,3}$(eV)	small QC	3592.06	3592.16	3592.13
	large QC	3591.46	3591.46	3591.52
$K\beta_{2,5}$(eV)	small QC	3601.97	3602.54	3603.54
	large QC	3601.97	3602.28	3603.08
[a]The calculated energy axis is shifted by 104.4 eV.				

Table S2. Löewdin Orbital Population						
orbital contribution (%)	$K\beta_{1,3}$			$K\beta_{2,5}$		
	KCl	KBr	KI	KCl	KBr	KI
K_{np}	94	73	98	6	6.5	6
K_{ns}	-	-	-	-	-	-
L_{ns}	4.4	25	0.8	0.2	0	-
L_{np}	0.6	0.6	0.4	93	93	94

Table S3. Experimental and Calculated energy of $K\beta_{1,3}$ and $K\beta_{2,5}$ Transitions[a]				
Electronic transition		KCl	KNO_3	K_2CO_3
$K\beta_{1,3}$(eV)	experimental	3589.9	3589.9	3589.8
	calculated	3592.1	3592.1	3592.2
$K\beta''$(eV)	experimental	-	-	3597.43
	calculated	-	3596.26	3597.47
$K\beta_{2,5}$(eV)	experimental	3601.97	3600.79	3601.9
	calculated	3601.97	3601.14	3601.9
[a]The calculated energy axis is shifted by 104.4 eV.				

**Table S4. Löewdin Orbital Population**									
orbital contribution (%)	K$\beta_{1,3}$			Kβ''			K$\beta_{2,5}$		
	KCl	KNO_3	K_2CO_3	KCl	KNO_3	K_2CO_3	KCl	KNO_3	K_2CO_3
K$_{np}$	94	98	97	8	2.0	1.9	7.2	2.1	2.4
K$_{ns}$	-	-	0	-	0.3	0.2	-	0	
L$_{ns}$	4.4	2	2	92	23.9	13.3	0.2	0	0
L$_{np}$	0.6	-	0	-	71.6	83.2	92.8	93.7	92.1

References

(1) Adiwidjaja, G.; Pohl, D. Superstructure of [alpha]-phase potassium nitrate. *Acta Crystallographica Section C* **2003**, *59*, i139-i140.

(2) Wolf, S.; Alam, N.; Feldmann, C. δ-KNO3: Synthesis and Structure of a New Modification of Potassium Nitrate. *Zeitschrift für anorganische und allgemeine Chemie* **2015**, *641*, 383-387.

(3) Gatehouse, B. M.; Lloyd, D. J. Crystal structure of anhydrous potassium carbonate. *Journal of the Chemical Society, Dalton Transactions* **1973**, 70-72.
